# Is there evidence for a noisy computation deficit in developmental dyslexia?

**DOI:** 10.3389/fnhum.2022.919465

**Published:** 2022-09-30

**Authors:** Yufei Tan, Valérie Chanoine, Eddy Cavalli, Jean-Luc Anton, Johannes C. Ziegler

**Affiliations:** ^1^CNRS, Laboratoire de Psychologie Cognitive (UMR 7290), Aix-Marseille University, Marseille, France; ^2^Institute of Language, Communication and the Brain, Aix-Marseille University, Aix-en-Provence, France; ^3^Laboratoire d’Étude des Mécanismes Cognitifs (EA 3082), Université Lumière Lyon 2, Lyon, France; ^4^CNRS, Institut des Neurosciences de la Timone (UMR 7289), Centre IRM-INT@CERIMED, Aix-Marseille University, Marseille, France

**Keywords:** dyslexia, neural noise, repetition, fMRI, neural adaptation

## Abstract

The noisy computation hypothesis of developmental dyslexia (DD) is particularly appealing because it can explain deficits across a variety of domains, such as temporal, auditory, phonological, visual and attentional processes. A key prediction is that noisy computations lead to more variable and less stable word representations. A way to test this hypothesis is through repetition of words, that is, when there is noise in the system, the neural signature of repeated stimuli should be more variable. The hypothesis was tested in an functional magnetic resonance imaging experiment with dyslexic and typical readers by repeating words twelve times. Variability measures were computed both at the behavioral and neural levels. At the behavioral level, we compared the standard deviation of reaction time distributions of repeated words. At the neural level, in addition to standard univariate analyses and measures of intra-item variability, we also used multivariate pattern analyses (representational similarity and classification) to find out whether there was evidence for noisier representations in dyslexic readers compared to typical readers. Results showed that there were no significant differences between the two groups in any of the analyses despite robust results within each group (i.e., high representational similarity between repeated words, good classification of words vs. non-words). In summary, there was no evidence in favor of the idea that dyslexic readers would have noisier neural representations than typical readers.

## Introduction

Fluent reading is a critical skill for personal and professional development in all modern societies (Beddington et al., 2008). Yet, a substantial portion of children have severe and long-lasting difficulties in learning to read despite conventional instruction, normal intelligence, and adequate socio-cultural opportunities (World Health Organisation [WHO], 2010). This neurodevelopmental disorder is called developmental dyslexia (DD), which affects between 5 and 17% of children (Snowling, 2000; Demonet et al., 2004; Norton et al., 2015).

A large number of theories have been proposed to explain the causes of DD, such as theories that highlight temporal deficits (Vandermosten et al., 2010; Goswami, 2011; Casini et al., 2018), auditory deficits (Boets et al., 2007), phonological deficits (Bradley and Bryant, 1978; Baldeweg et al., 1999; Helenius et al., 1999; Snowling, 2001; Ramus et al., 2003; Ziegler et al., 2009), attentional deficits (Facoetti et al., 2000, 2006, 2008), visual deficits (Stein and Walsh, 1997; Stein, 2014), or cerebellar deficits (Nicolson et al., 2001; Nicolson and Fawcett, 2005). There is some consensus that no single-deficit theory can currently explain the multifactorial nature of the deficits observed in DD (Pennington, 2006; Perrachione et al., 2016; Ziegler et al., 2019; O’Brien and Yeatman, 2021). Thus, a more general theory is needed to explain the variety of sensory deficits associated with DD.

One elegant theory that has the potential to explain the various facets of DD is the neural noise hypothesis (Hancock et al., 2017). Neural noise can be defined as a stochastic variability in the neural response to repeated presentations of the same stimulus. For example, a neuron that spikes at widely variable intervals in response to repeated stimulus presentations is considered to be noisier than one that spikes at nearly the same time following each presentation. The link with DD seems rather straightforward: an excessive amount of neural noise impairs the capacity of populations of neurons to maintain stable patterns of activity, which is detrimental to both forming and maintaining representations.

How can one explain that excessive amounts of neural noise specifically affect learning-to-read more than other language processes? It could be argued that in most language processing situations, our brains are used to “cleaning-up” partial or incorrect bottom-up information using context (Pitt and Samuel, 1995). This is different in a learning-to-read situation. In the initial stages of reading acquisition, children have to learn the mapping between isolated graphemes and phonemes to set up the decoding network (Ziegler and Goswami, 2005; Ziegler et al., 2014, 2020). In this situation, “noisy” letter or phoneme information is detrimental because the same letter needs to map onto a single stable phoneme representation (B- > /b/and not/p/). Indeed, it has been shown in computational modeling that small amounts of noise in phoneme representations quickly result in catastrophic learning (Harm and Seidenberg, 1999; Ziegler et al., 2014, 2020; Perry et al., 2019). Similarly, small amounts of noise will also prevent the reading system from creating stable visual representations of letters and words, which will impair orthographic learning (Ziegler et al., 2014).

Strong evidence for the neural noise theory comes from studies that investigated the consistency of auditory brainstem responses to speech syllables in normal hearing children with a wide range of reading abilities (Hornickel et al., 2009; Hornickel and Kraus, 2013). The auditory brainstem response to speech closely mimics the spectrotemporal features of the stimulus. Hornickel and Kraus (2013) found that poor readers have significantly more variable auditory brainstem responses to speech than do good readers, independent of resting neurophysiological noise levels. Liebig et al. (2020) have shown that the neural stability of the auditory brainstem response to isolated syllables (e.g., /da/) measured at kindergarten predicted reading and spelling performance 2 years later.

One way to measure neural noise is through stimulus repetition. When neural noise is excessive, the neural encoding of repeated items should be more variable and neural adaptation to repeated items should be reduced. Excessive neural noise might make it difficult to establish robust short-term perceptual representations which are the basis for neural adaptation effects (Garrido et al., 2009). Indeed, Perrachione et al. (2016) showed that adults and children with dyslexia exhibited significantly diminished neural adaptation for a wide variety of repeated stimuli (spoken words, written words, visual objects, and faces). Similarly, Gertsovski and Ahissar (2022) showed no neural adaptation to repeated sounds in auditory cortex and other higher-level regions in adults with dyslexia compared to typically developing readers. This might also explain why individuals with dyslexia may have an impairment “anchoring” to consistent stimulus statistics in order to exploit sensory history for learning (Ahissar et al., 2006; Ahissar, 2007; but see Ziegler, 2008). Zhang et al. (2021) used electroencephalography (EEG) with frequency-tagging to track the temporal evolution of speech-structure learning (a structured vs. a random stream of repeated tri-syllabic pseudowords) in children with dyslexia and found that the learning of implicit speech structures built up more slowly in children with dyslexia than in typically developing readers. Studies in the visual domain reported slower perceptual decision making in individuals with dyslexia (Stefanac et al., 2021; Manning et al., 2022), which may be related to excessive perceptual noise. However, not all studies reported greater neural variability to be associated with poorer reading. In particular, Malins et al. (2018) showed a positive relationship between trial-by-trial activation variability in the left inferior frontal gyrus (IFG) pars triangularis and reading skill suggesting that greater levels of neural variability were associated with better reading skills.

A few studies focused on the effects of repetition and prediction rather than neural variability or neural adaption. Using an odd-ball paradigm, Beach et al. (2022b) recorded magnetoencephalography (MEG) as adults with and without dyslexia were passively exposed to speech syllables. In both groups, standards generated by as few as two repetitions were distinct from deviants, indicating normal sensitivity to repetition in dyslexia. However, only in the control group did standards become increasingly different from deviants with repetition. In another study Beach et al. (2022a) focused on prediction errors by presenting repeated words or faces with a high probability of stimulus repetition vs. a high probability of stimulus change. They found that the neural prediction error (as measured by EEG) was significantly weaker in dyslexia than the control group for both faces and words. These results were taken to suggest that “many of the mechanisms that give rise to neural adaptation as well as mismatch responses are intact in dyslexia, with the possible exception of a putatively predictive mechanism that successively integrates recent sensory information into feedforward processing” (Beach et al., 2022b, p. 1). Finally, Pugh et al. (2008) used an animacy judgment task (living/non-living) to investigate the effects of stimulus repetition in normal and dyslexic readers in functional magnetic resonance imaging (fMRI). In every block, six words were repeated six times in a pseudorandom fashion and intermixed with 20 unrepeated words that served as unrepeated control words. Their results showed that repetition had a similar (facilitatory) effect on reaction time and accuracy for both normal and dyslexic readers. In the critical regions of the left-hemisphere reading network, typically developing readers showed reduced activation for repeated words while dyslexic readers showed increased activation with repetition in these same reading-related sites, suggesting that the left-hemisphere reading circuitry in adolescent dyslexics is poorly tuned but not wholly disrupted.

In the present study, we wanted to test the neural noise theory more directly by investigating whether behavioral and neural responses to repeated words are more variable across repetitions for dyslexic readers. As suggested by Hancock et al. (2017), “systems-level multimodal imaging studies that measure response variability in reading disorders, such as using phase locking measures in EEG or single-trial estimates of BOLD response (…), can provide a direct test of the basic premise of our hypothesis” (p. 445). We followed their suggestion by measuring single-trial estimates of BOLD response in an fMRI experiment for 36 words that were repeated 12 times both for a group of dyslexic and typical readers. The present study was conducted with adult dyslexics who all had a history of childhood dyslexia and were referred to us from a regional clinical reference center of dyslexia with a formal diagnosis of dyslexia. Although one could argue that testing adult university students with dyslexia is suboptimal because the neural noise deficit might have been compensated for, we believe that one can make the opposite case. That is, reading compensation strategies might affect reading outcomes but they should not alleviate neural noise. Thus, it seems to be a fair comparison to investigate neural noise differences in groups that no longer show massive behavioral differences.

**FIGURE 1:**
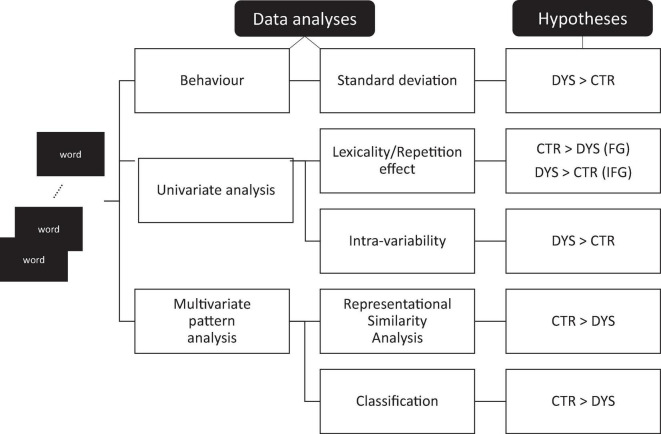
Testing the neural noise theory. Main predictions of the neural noise theory for the various analyses. DYS: dyslexic readers, CTR: typical readers, FG: fusiform gyrus, IFG: inferior frontal gyrus.

Variability measures were computed for the two groups both at the behavioral and neural level. At the behavioral level, we compared the standard deviation of reaction time distributions when participants read aloud the same set of words 12 times. At the neural level, we first looked at standard univariate (whole-brain) analyses and univariate region of interest analysis. In line with the neural adaptation results (Perrachione et al., 2016), we expected to find that the key regions of the reading system should be less sensitive to repetition in adults with DD than typical readers. We then looked at measures of intra-item variability in the BOLD signal to repeated words. That is, we compared the standard deviation of the beta values for multiple repetitions of the same words between the two groups. If the brain responses to repeated items were noisier in DD, dyslexic readers would show greater amounts of variability than typical readers. We then used multivariate pattern analyses, namely representational similarity (Kriegeskorte et al., 2008) and multivariate pattern classification (Pisner and Schnyer, 2020) to further explore the predictions of the neural noise hypothesis. Again, the logic was straightforward: If neural representations of written words were noisier for adults with DD, neural similarity between repeated words should be reduced in adults with DD. Similarly, if neural responses to repeated words were noisier in adults with DD, a classifier that was trained to discriminate words from hash marks should perform less well for adults with DD than for typical readers. The various predictions of the study are illustrated and summarized in Figure 1.

## Materials and methods

### Participants

Twenty adults with dyslexia and 20 skilled readers aged between 20 and 29 years participated in the present study. All participants were university students and native speakers of French. They were recruited at Aix-Marseille University (France) from a wide variety of academic programs (i.e., in each group, 55% of the participants were enrolled in social science programs and 45% were enrolled in science programs). The university students with dyslexia were recruited through a national clinical reference center of learning disorders (*Centre de Référence des Troubles des Apprentissages* at the Salvator Hospital in Marseille) and the *Mission Handicap* (University Medical Service) of Aix-Marseille University. They were all diagnosed with dyslexia in primary school and had received remedial teaching for an average of 5.34 years (SD = 0.41). Furthermore, they reported having struggled with reading from childhood to adulthood. The group of dyslexic readers (DYS) and the group of typical readers (CTR) were matched on gender (DYS: 9 females and 11 males; CTR: 11 females, 9 males), chronological age [DYS = 22.95 ± 2.56, CTR = 23.45 ± 2.42, t(38) = −0.64, *p* = 0.53], verbal IQ [DYS = 38.20 ± 5.15, CTR = 39.58 ± 4.26, t(38) = −1.11, *p* = 0.28], and non-verbal IQ [DYS = 41.60 ± 8.33, CTR = 42.20 ± 7.45, t(38) = −0.24, *p* = 0.81]. The study conforms to recognized standards of the World Medical Association Declaration of Helsinki and was approved by the National Ethics Committee for Biomedical Research. All participants gave written informed consent and received €50 for their participation.

### Stimuli and procedure

#### Reading level assessment

The reading level of the participants was assessed with two standardized reading tests. The first was the Adult Reading History Questionnaire (ARHQ, Lefly and Pennington, 2000), which is a self-report questionnaire used to diagnose the history of reading difficulties, which includes items on reading habits, reading and spelling abilities, reading speed, attitudes toward school and reading, additional assistance received, repeating grades or courses and effort required to succeed, separately from elementary school, secondary school, post-secondary education, and current life (Deacon et al., 2012). Participants answer each item on a 5-point Likert-type scale ranging from 0 to 4. The total score is divided by the maximum possible score (92), resulting in a proportion score ranging from 0 to 1. Higher scores indicate greater reading difficulties. Norms are available from an adult sample of 1,107 participants (Fichten et al., 2014). In addition, they performed the Alouette reading test (Lefavrais, 2005), which is a sensitive standardized reading fluency test for adults with dyslexia (Cavalli et al., 2017a) with excellent psychometric properties (Bertrand et al., 2010). Norms are available from an adult sample of 164 participants (Cavalli et al., 2017a). The critical variable was reading efficiency (CTL) using the following equation: CTL = A × 180/RT, where A is the number of words correctly read (self-corrections included) and RT is the reading time.

#### Reading aloud task (in scanner)

For the reading aloud task, we selected 34 French words with frequencies ranging from 1 to 125.8 per million (Mean = 26.03, SD = 38.37) (New et al., 2004), and lengths ranging from 2 to 10 letters (Mean = 6.79, SD = 1.70). The words were presented in 40-point, Arial font. While 34 hash mark combinations were used as baseline, the number of each hash mark combination was matched with the length of each word. All stimuli were presented in white against a black background and subtended about 1.4° of visual angle for each letter.

**FIGURE 2:**
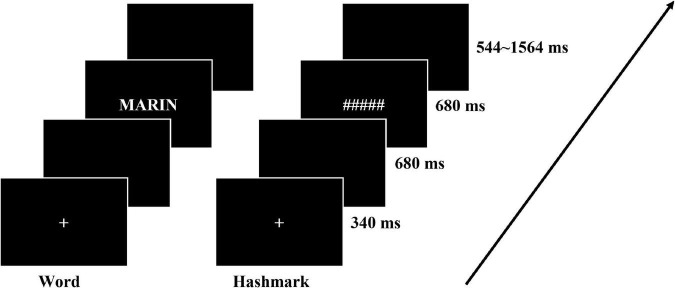
Illustration of the experimental procedure and timing in the reading aloud task that was performed in the scanner.

The stimuli were presented using an in-house software developed in the NI LabVIEW environment (Bitter et al., 2017). The software was launched and real-time synchronized with the MR acquisition using a NI-PXI 6289 digital input/output hardware, which also allowed vocal and motor answers recording. The participants lied in the MRI scanner and the stimuli were projected through a mirror onto a screen (1024 × 768), the 768 × 768 square field of view covered a 20° FOV angle. Each trial started with a fixed cross presented at the center of the screen for 340 ms, after a blank of 680 ms, a word was displayed for 680 ms, and the participants were instructed to read the words aloud while ignoring the hash marks (see Figure 2). The inter-trial interval jittered from 544 to 1,564 ms. There were 4 runs for each participant, each run was composed of 136 trials made of 34 words repeated three times and 34 hash mark combinations. The trials in each run were presented pseudo randomly and the order of 4 runs was counterbalanced across participants. Along with the fMRI signal, participants’ answers were recorded together using the FOMRI-II microphone (Optoacoustics Ltd., Or-Yehuda, Israel).

#### Localizer task (in scanner)

The localizer task was adapted from a 5-min fast acquisition procedure designed by Pinel et al. (2007), which has shown to successfully activate the brain regions of auditory and visual perception, motor system, reading, language comprehension and mental calculation at an individual level. Eight types of stimuli were used: flashing horizontal checkerboards, flashing vertical checkerboards, visual motor instructions, auditory motor instructions, visual sentences, auditory sentences, visual subtraction, auditory subtractions. Each type of stimuli was presented in 10 successive trials in a randomized order for each participant. Flashing checkerboards were presented for 136 ms on each trial and participants were instructed to passively view the checkboards. For visual stimuli, each trial was composed of four successive screens of 272 ms and participants gave their responses by pressing the left or right button. For auditory stimuli, each trial lasted between 2,040 and 3,672 ms and participants also gave their responses by pressing the left or right button. The inter-trial interval ranged from 408 to 6,528 ms. The presentation of visual and auditory stimuli and the recording of responses were done in the same way as in the reading aloud task.

### Data acquisition

The MRI data were acquired on a SIEMENS MAGNETOM Prisma 3T scanner with a 64-channel head coil at the Centre IRM-INT@CERIMED (INT, UMR 7289 CNRS–Aix-Marseille University). The functional images were acquired using a T2*-weighted gradient-echo planar sequence with 54 slices per volume [repetition time (TR) = 1.224 s, echo time (TE) = 30 ms, multi-band acceleration factor = 3, voxel size = 2.5 mm × 2.5 mm × 2.5 mm, flip angle = 65°, field of view (FOV) = 210 mm × 210 mm, matrix size = 84 × 84]. The anatomical image was acquired using high-resolution structural T1-weighted image with 256 slices (TR = 2.4 s, TE = 2.28 ms, voxel size = 0.8 mm × 0.8 mm × 0.8 mm, flip angle = 8°, FOV = 256 mm × 256 mm, matrix size = 320 × 320). A fieldmap acquisition (54 slices per volume, TR = 7.06 s, TE = 59 ms, voxel size = 2.5 mm × 2.5 mm × 2.5 mm, flip angle = 90°, FOV = 210 mm × 210 mm, matrix size = 84 × 84) was collected to estimate and correct the B0 inhomogeneity. A total of 1096 functional scans were acquired over 4 runs (5.59 min per run) for the reading task, and 256 functional scans were acquired in one run (5.22 min) for the localizer task.

### Data analyses

#### Behavioral data analysis

Because the reading aloud responses were recorded in the scanner, the wave files had to be denoised in order to determine the onset time. Denoising was performed using the Wavelet Signal Denoiser toolbox of Matlab.^1^ We then used the Praat software (Boersma, 2001) to determine the onset of each reading aloud response (RT) and judge whether the word pronounced correctly. The data from one participant was excluded from the behavioral data analysis because of missing data.

Then, we calculated the mean accuracy and mean RT of each group. We used a two-factorial analysis of variance (ANOVA) with group (DYS vs. CTR) and repetition (12 repetitions) as factors to test for significant differences between the two groups. The assumption of sphericity was checked by Mauchly’s test, and if it was violated, the Greenhouse–Geisser correction was used to correct the *F*-test results.

The standard deviation of the RT distribution across 12 repetitions for each word was measured to analyze the variability, and a two-sample *t*-test was conducted to analyze the differences in RT variability between the two groups.

#### Univariate analysis of the functional magnetic resonance imaging data

##### Whole-brain analysis

The fMRI data was preprocessed and analyzed using SPM12 software (Wellcome Institute of Cognitive Neurology, London, UK).^2^ First, we used the fieldmap images to measure field inhomogeneities, then the functional (EPI) images were realigned using the fieldmap for distortion and motion correction. The anatomical (T1) images were coregistered to the mean image of realigned EPI images. The coregistered T1 image was segmented into Gray Matter (GM), White Matter (WM), CerebroSpinal Flux (CSF), Bone tissue and Soft tissue, and normalized into standard Montreal Neurological Institute (MNI). Finally, the realigned EPI images were also normalized into MNI space using the deformation field image obtained during the anatomical normalization process, and spatially smoothed with a 5 mm full-width at half-maximum (FWHM) isotropic Gaussian kernel. The explicit masks included GM, WM, and CSF.

Prior to the first-level analysis, EPI images were denoised by GLMdenoise toolbox (Kay et al., 2013). The functional data in the first-level models were high pass filtered with a cut-off of 128 s and corrected for autocorrelation by an autoregressive model of order 1. A general linear model (GLM) in SPM12 was used to estimate the parameters. There were six regressors for the experimental conditions in each run, three for word repetitions and three for hash mark repetitions, and one regressor for runs. The duration of each event was 1.222 s. The onset and duration of each stimulus were convolved with the canonical hemodynamic response function and modeled as regressors in the design matrix.

T-contrast maps were computed separately for the lexicality effect and the word repetition effect using a voxel-based random effect analysis (RFX). The lexicality effect was obtained by subtracting activation in the control condition (hash marks) from activation in the word condition. The word repetition effect was measured by identifying regions exhibiting a change in BOLD responses across three repetitions in each of the four runs that fit a linear function (i.e., 1 × 1st repetition, 0 × 2nd repetition, −1 × 3rd repetition). The contrast maps from the first-level analyses were used to conduct the second-level one-sample *t*-test to test for significant group differences for the two effects. The activation areas were labeled using the Anatomical Automatic Labeling (AAL) package (Tzourio-Mazoyer et al., 2002).

##### Univariate region of interest analysis

For the univariate analysis on ROIs, we chose eight anatomical ROIs that are typically reported in studies of normal and impaired reading (Paulesu et al., 2014; Martin et al., 2015; Rueckl et al., 2015): left and right IFG, left and right fusiform gyrus (FG), left and right angular gyrus (AG), and left and right supramarginal gyrus (SMG). In addition, we used dorsal extrastriate cortex (hOC3d) as a purely visual control area (Kujovic et al., 2013). These ten anatomical ROIs were generated from SPM Anatomy toolbox (Eickhoff et al., 2005) and the WFU_PickAtlas.^3^ They are shown in the Supplementary Figure 1.

The preprocessing was similar to the univariate whole-brain analysis. However, the images were not spatially normalized or smoothed to take advantage of high spatial-frequency pattern information within each participants’ data (Kriegeskorte et al., 2006). They were also denoised using the GLM Denoise toolbox. All ROIs were converted into the native space of each participant using the inverse of the transformation matrix that was used to normalize the T1 image into the standard MNI space.

For a given ROI mask, we extracted each subject’s percent signal change^4^ using “mean” calculation across voxels. For each effect (“Lexicality” and “Repetition”), we obtained a matrix of percent signal changes per subject (*n* = 20) and per ROI (*n* = 10). The outliers (values that were greater than 2.5 standard deviations above or below the median) in a given ROI were replaced by the mean computed across subjects.

For each ROI, we performed one-tailed permutation tests^5^ to compare the distribution of the percent signal changes of a given condition (“Lexicality” or “Repetition”) to the null hypothesis (normal distribution) within a group of subjects or between the two groups. Statistical tests were conducted using 2000 permutations and two types of multiple comparisons, False Discovery Rate (FDR, Benjamini and Hochberg, 1995) and Bonferroni’s (Bland and Altman, 1995).

##### Intra-item variability

For the intra-item variability analysis, preprocessing was identical to the univariate ROI analysis. The same words were repeated three times in each run. To make a reasonable comparison with hash marks that were always the same except that they varied in length, we selected five hash marks that repeated more than three times per run and we extracted only the first three repetitions in each run. The activation of each single trial (34 words × 3 repetitions and 5 hash marks × 3 repetitions for 4 runs, 468 trials in total) was estimated using the Least Squares All (LSA) model (all trials are estimated simultaneously in a single model) following the methodology of Mumford et al. (2014). A GLM in SPM12 was used to estimate the parameters. There were 117 regressors in each run, including 102 regressors for each word and 15 regressors for each hash mark, and also one regressor for runs. The duration of each event was 1.222 s. The onset and duration of each stimulus were convolved with the canonical hemodynamic response function and modeled as regressors in the design matrix. We therefore obtained 468 beta maps for each participant.

The individual and functional regions of interest (ROIs) were obtained from the localizer task. Because the functional data were not normalized or smoothed, we did the same for the localizer data. Participant specific contrasts of “reading sentences” vs. “flashing checkboards” were calculated to identify the reading network of each participant (Pinel et al., 2007). Only the voxels that were active at a voxel-wise statistical threshold of *p* < 0.001 (without correction for multiple comparisons) were included in the individual functional ROI. The 10 anatomical ROIs were the same as those used in the univariate ROI analysis. We then extracted the data from all the images masked with each ROI for each participant.

Given that each word was repeated for three times in four runs, we had 12 masked beta maps for each word. Because some of the beta values of some voxels were outliers, we replaced these extreme values (beta values greater than 2.5 standard deviations above or below the mean) in a masked beta map by the mean beta value of all voxels. We then calculated the standard deviation of the 12 beta maps voxel by voxel in a given ROI for each word, then averaged all the standard deviations in this ROI to obtain the mean variability of each word. Finally, the mean variabilities of the 34 words were averaged to obtain the mean variability of each participant. In order to compare the mean variability between the dyslexic and control groups, we performed a two-sample *t*-test. These analysis steps were repeated for each ROI.

#### Multivariate pattern analysis for functional magnetic resonance imaging data

##### Representational similarity analysis

For the representational similarity analysis, the preprocessing and first-level analysis of the fMRI data were the same as the intra-item variability analysis. We also used the same functional localizer ROI and the same anatomical ROIs. Thus, the analysis was based on 12 masked *t* maps for each word and hash mark.

The representational dissimilarity matrix (RDM) was obtained by measuring the correlation distance between each pair of conditions, i.e., 1 min the linear correlation between neural patterns of two conditions (Haxby et al., 2001; Aguirre, 2007; Kiani et al., 2007) which characterizes the dissimilarity between different activity patterns. In our case, we wanted to know if neural similarity between repeated words in adults with DD would be weaker than in typical readers. Thus, we measured the correlation distance between each word repetition, which resulted in a 12 × 12 repetition representational dissimilarity matrix for each word. The RDM of each word across the 12 repetitions within a given ROI was calculated using the CosMoMVPA toolbox (Oosterhof et al., 2016). In order to reduce the influence of the differences across runs, the RDM was subtracted from the mean of the entire matrix. Then the demeaned RDMs of all the words were averaged to get the mean RDM of each subject. For the statistical group analysis, we averaged all the values in the lower triangular part of the mean RDM leaving out the diagonal as the mean dissimilarity of 12 word repetitions for each subject. A two-sample *t*-test was performed to assess the differences between the dyslexic and the control groups. These analysis steps were repeated for each ROI.

##### Support vector machine classification of words and hash marks

For the classification analysis, preprocessing of fMRI data was the same as before, i.e., the images were not spatially normalized or smoothed. The explicit masks used for the statistical analysis included gray matter, white matter, and cerebrospinal flux.

The first-level analysis was different from the other analysis, because we used all the hash marks here. The activation of each single trial (34 words × 3 repetitions and 34 hash marks for 4 runs, 544 trials in total) was estimated using the Least Squares All (LSA) model (all trials are estimated simultaneously in a single model). A GLM in SPM12 was used to estimate the parameters. There were 136 regressors for all experimental conditions in each run, including 102 regressors for each word and 34 regressors for each hash mark, and also one regressor for runs. The duration of each event was 1.222 s. The onset and duration of each stimulus were convolved with the canonical hemodynamic response function and modeled as regressors in the design matrix. T-contrast maps were computed separately for each trial using a voxel-based random effect analysis (RFX). We therefore obtained 544 *t*-contrast maps for each participant.

We used Nilearn,^6^ a Python package of machine learning for neuroimaging data (Pedregosa et al., 2011) to classify words and hash marks for each participant. We used supervised learning and cross-validation. That is, the model was first trained with the labeled data and then tested on new unlabeled data to predict the labels. A Support Vector Machine (SVM) classifier with linear kernel was used to learn associations between data patterns and labels. In order to avoid overfitting, fourfold cross-validation was used to split data into training sets and testing sets. Because our data were imbalanced in the distribution of the target classes (408 words vs. 136 hash marks), Stratified Shuffle Split iteration was used to ensure that relative class frequencies were approximately preserved in each train and validation fold. Stratified Shuffle Split can create splits by preserving the same percentage for each target class as in the complete set. Classification performance was quantified by measuring the area under the receiver operating characteristic (ROC) curve (i.e., ROC-AUC score), which avoids inflated performance estimates for imbalanced datasets.

Permutation testing was used to evaluate the significance of the cross-validated score. The *p*-value approximates the probability that the score would be obtained by chance. It is calculated as (C + 1)/(n_permutations + 1), where C is the number of permutations whose score is greater than or equal to the true score. The n-permutation was set to 1000. Thus, the best possible *p*-value is 1/(n_permutations + 1) = 0.00099 and the worst is 1.0. We then performed a two-sample *t*-test to compare the ROC-AUC scores for the two groups.

## Results

### Reading level assessment

As expected, the results on the ARHQ showed that the ARHQ score of the dyslexic group was significantly higher than that of the control group [DYS = 0.58 ± 0.08, CTR = 0.33 ± 0.08, Cohen’s *d* = 3.12, *t*(38) = 9.57, *p* < 0.001]. The results of the standardized reading test (Alouette) showed that dyslexic group obtained significantly lower scores than the control group [DYS = 368.79 ± 73.00, CTR = 493.03 ± 60.73, Cohen’s *d* = −1.85, *t*(38) = −5.85, *p* < 0.001]. When compared to the published norms of these two tests, the scores of our sample of dyslexic readers were 2.0 standard deviations above the published norms on the ARHQ (Fichten et al., 2014) and 2.2 standard deviations below the published norms the Alouette test (Cavalli et al., 2017a).

### Reading aloud task (in the scanner)

**FIGURE 3:**
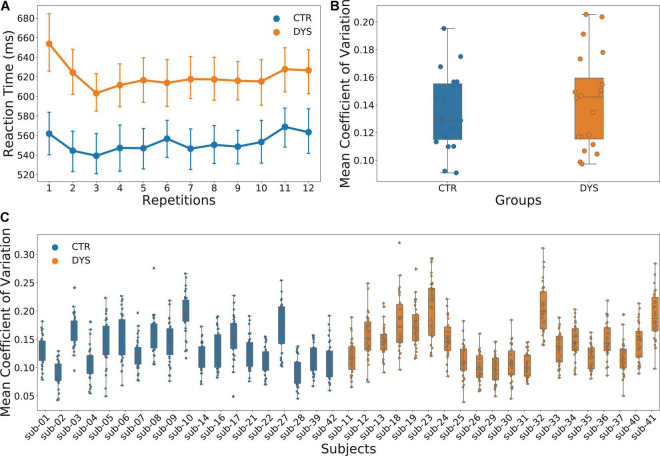
Results of reading aloud latencies. **(A)** Mean reaction times (RTs) across the twelve repetitions for both typically developing (CTR) and dyslexic readers (DYS), **(B)** scattered box plots of mean coefficients of variation of RTs for both CTR and DYS, **(C)** scattered box plots of mean coefficients of variation of RTs calculated for each word across the 12 repetitions and for each participant.

The mean accuracy was at ceiling with 99.9% for the controls and 99.4% for the dyslexic readers. We therefore analyzed only reading aloud latencies (RTs). For each participant, outliers with 2.5 standard deviation above and below the mean RT were deleted. There was no significant difference in the number of outliers between two groups [DYS = 8.65 ± 2.87, CTR = 8.74 ± 3.26, *t*(37) = 0.09, *p* = 0.93]. The mean RT for the dyslexic group was 621 ms and that for the control group was 552 ms. The results of ANOVA showed a significant main effect of group [*F*_(1, 37)_ = 5.373, *p* = 0.026] and a significant main effect of repetition [*F*_(11, 407)_ = 2.863, *p* = 0.001]. However, the interaction effect between group and repetition was not significant [*F*_(11, 407)_ = 0.810, *p* = 0.630]. The results are plotted in Figure 3A. Because the strongest repetition effects were obtained in the first three repetitions (i.e., the first run), we repeated the ANOVA with the first three repetitions only. The results were identical to the previous analysis with a significant main effect of group [*F*_(1, 37)_ = 6.143, *p* = 0.018], a significant main effect of repetition [*F*_(2, 74)_ = 9.906, *p* = 0.001] and no significant interaction between the effects of group and repetition [*F*_(2, 74)_ = 1.419, *p* = 0.248].

In order to analyze the differences in RT variability across the two groups, we calculated the standard deviation across the 12 repetitions for each word. However, because the mean RTs of two groups were significantly different, we used the coefficient of variation (i.e., the ratio of the standard deviation to the mean) instead of the standard deviation across the 12 repetitions for each word. We then averaged the coefficients of variation across the 12 repetitions of each item for each participant. As can be seen in Figure 3B, there was no significant difference between two groups on the mean coefficient of variation [t(38) = −0.599, *p* = 0.553]. The results for each participant are shown in Figure 3C. In sum, there is no evidence for greater variability for repeated words in adults with dyslexia as compared to typical readers.

### Univariate analysis of the functional magnetic resonance imaging data

#### Whole-brain analysis

We first analyzed whether there were any differences between the two groups in response to words. We therefore contrasted words against hash marks for each participant using a voxel-based random effect analysis (RFX). We then performed a one-sample *t*-test to obtain the mean activation of each contrast in each group, which were labeled using the Anatomical Automatic Labeling (AAL) package. Finally, we compared the activation of each contrast between two groups with a two-sample *t*-test. All reported results use an uncorrected voxel-wise statistical threshold of *p* < 0.001, and a cluster-wise threshold of *p* < 0.05 corrected for multiple comparisons over the whole brain. Correction for multiple comparisons was based on Random Field Theory as implemented in the SPM12 software (Nichols, 2012).

**FIGURE 4:**
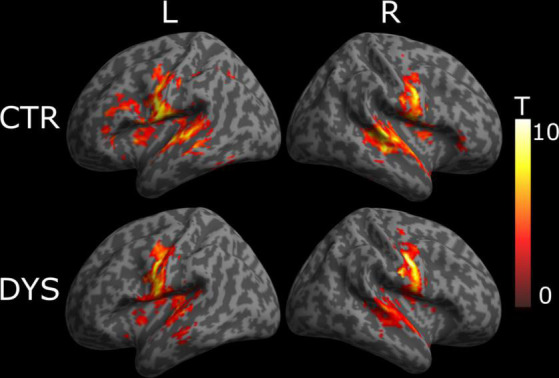
Univariate whole-brain results of the Lexicality effect (words minus hash marks). Statistical t-maps for dyslexic readers (DYS, *n* = 20) and controls (CTR, *n* = 20) are projected on **left (L)** and **right (R)** cortical surfaces (from MNI standard human cortex) using an uncorrected voxel-wise threshold of *p* < 0.001 and a cluster-wise threshold with FDR correction of *q* < 0.05.

For typical readers, we found word-specific activation mainly in bilateral superior temporal gyrus, left post-central gyrus, left thalamus, right rolandic operculum, right middle temporal gyrus, and right cerebellum (see the upper part of Figure 4). For dyslexic readers, we found word-specific activation mainly in bilateral superior temporal gyrus, bilateral precentral gyrus, left putamen, right pallidum and bilateral cerebellum (see the lower part of Figure 4). The full list of activation clusters is presented in Supplementary Tables 1, 2 for controls and dyslexic readers, respectively. The two-sample *t*-test did not show any significant differences between the two groups (at a voxel-wise statistical threshold of *p* < 0.001 without correction, and a cluster-wise threshold of *q* < 0.05 with FDR correction).

**FIGURE 5:**
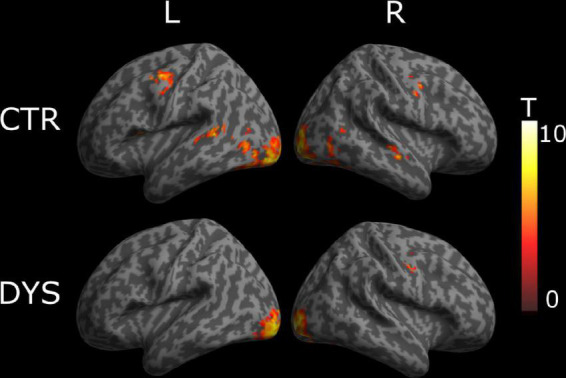
Univariate whole-brain results of the deactivation/repetition effect (a decreased activation across repetitions that fit a linear function). Statistical t-maps for dyslexic readers (DYS) and controls (CTR) are projected on **left (L)** and **right (R)** cortical surfaces (from MNI standard human cortex) using an uncorrected voxel-wise threshold of *p* < 0.001 and a cluster-wise threshold with FDR correction of *q* < 0.05.

We then analyzed whether dyslexic readers showed weaker repetition (deactivation) effects than controls. For this purpose, we calculated repetition effect contrasts, which identify regions exhibiting a change in BOLD responses across three repetitions that fit a linear function. The results are shown in Figure 5. In typical readers, we found a linear deactivation/repetition effect in left precentral gyrus, bilateral lingual gyrus, bilateral inferior temporal gyrus, right middle occipital gyrus, left cerebellum, left inferior temporal gyrus and left middle temporal gyrus (see the upper part of Figure 5). For dyslexic readers, we found a linear deactivation/repetition effect in right lingual gyrus, left inferior occipital gyrus, left middle occipital gyrus, left fusiform gyrus, left cerebellum (see the lower part of Figure 5). The full list of activation clusters is presented in Supplementary Tables 3, 4 for controls and dyslexic readers, respectively. The results of a two-sample *t*-test showed no significant differences between the two groups (at an uncorrected voxel-wise statistical threshold of *p* < 0.001, and a cluster-wise threshold of *q* < 0.05 with FDR correction).

#### Univariate region of interest analysis

We performed an additional univariate ROI analysis using the 10 ROIs mentioned before. Because the behavioral data showed larger repetition effect in the first run, we focused on a ROI analysis of the first run to make sure we would not miss a potential effect. The analysis for all runs is found in the Supplementary material.

**FIGURE 6:**
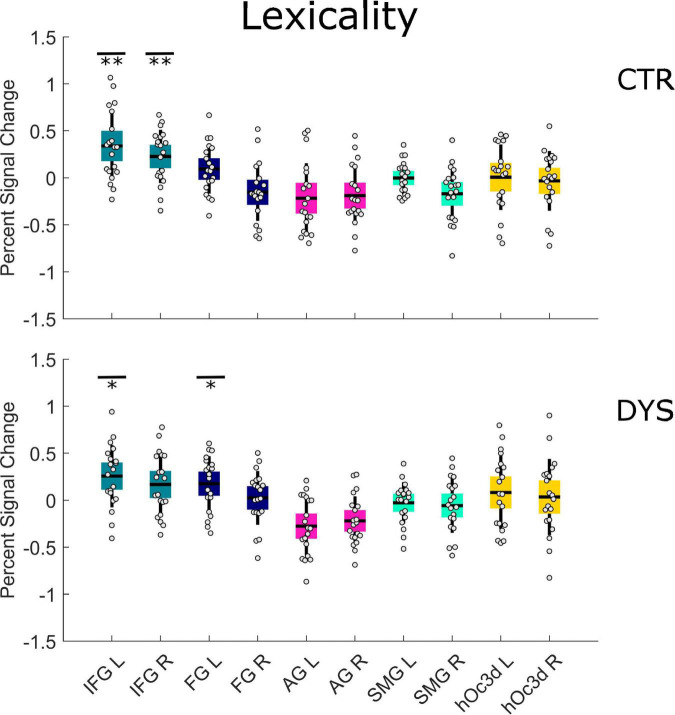
Univariate ROI results of the Lexicality effect (words minus hash marks). Percent signal change is displayed for each group (DYS and CTR) for each of the 10 predefined ROIs. The asterisks indicate significant effects *p < 0.05 or **p < 0.01 after correcting for multiple comparisons using FDR. DYS: dyslexic readers, CTR: typical readers, IFG: inferior frontal gyrus, FG: fusiform gyrus, AG: angular gyrus, SMG: supramarginal gyrus, hOc3d: Dorsal extrastriate cortex, L: left hemisphere, R: right hemisphere.

For the first run, we found a significant lexicality effect in the left IFG (*p* = 0.005 with FDR correction) and the right IFG (*p* = 0.005 with FDR correction) for the control group (see the upper part of Figure 6 and Supplementary Table 5). For the dyslexic group, we found a significant lexicality effect in the left IFG (*p* = 0.015 with FDR correction) and the left FG (*p* = 0.037 with FDR correction) (see the lower part of Figure 6 and Supplementary Table 5). A two-sample *t*-test showed that dyslexic readers exhibited a significantly greater lexicality effect than typical readers only in the right FG (*p* = 0.033 uncorrected) (see the Supplementary Table 5).

**FIGURE 7:**
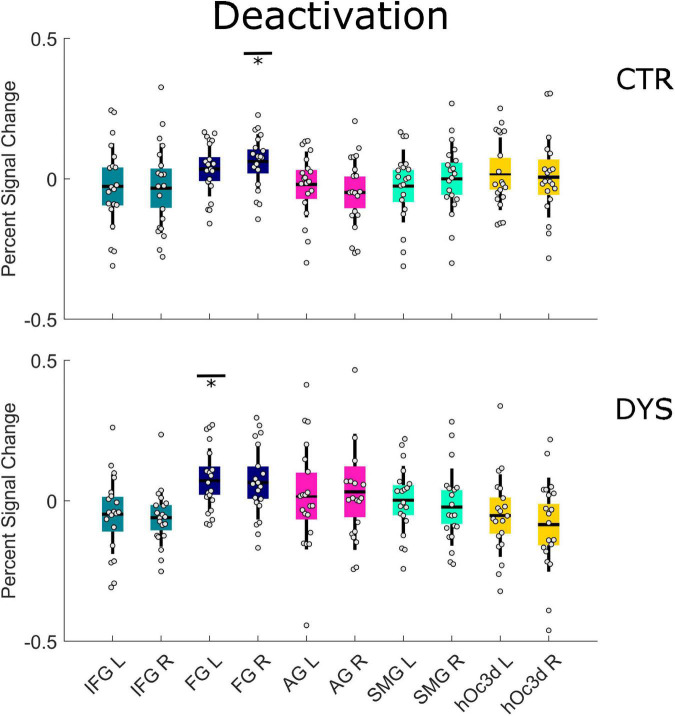
Univariate ROI results of the deactivation repetition effect (regions that show a linear decrease in activation). Percent signal change is displayed for each group (DYS and CTR) and for each of the ten predefined ROIs. The asterisks indicate significant effects *p < 0.05 after correcting for multiple comparisons using FDR. DYS: dyslexic readers, CTR: typical readers, IFG: inferior frontal gyrus, FG: fusiform gyrus, AG: angular gyrus, SMG: supramarginal gyrus, hOc3d: Dorsal extrastriate cortex, L: left hemisphere, R: right hemisphere.

As concerns the repetition deactivation effect, we found a significant deactivation in the right FG (*p* = 0.030 with FDR correction) for the control group (see the upper part of Figure 7 and Supplementary Table 6) and in the left FG (*p* = 0.050 with FDR correction) for the dyslexic group (see the lower part of Figure 7 and Supplementary Table 6). There was no significant difference between the two groups in any of the ROIs (see the Supplementary Table 5). The results for all runs were similar to those of the first run (see Supplementary Tables 7, 8).

#### Intra-item variability

**FIGURE 8:**
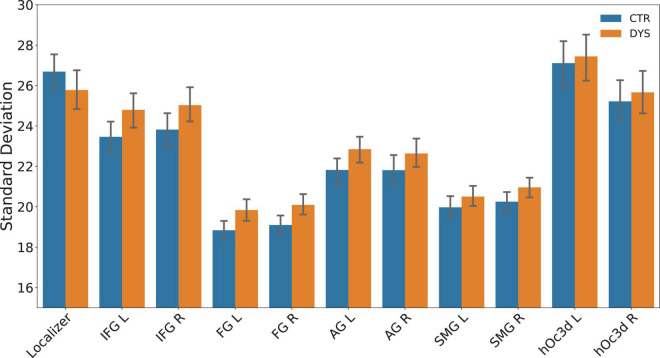
The mean intra-word variability (mean SD) across the 12 repetitions of beta values in each ROI for the control (CTR) and dyslexic (DYS) group. IFG: inferior frontal gyrus, FG: fusiform gyrus, AG: angular gyrus, SMG: supramarginal gyrus, hOc3d: dorsal extrastriate cortex, L: left hemisphere, R: right hemisphere.

Because each item was repeated three times in each of the four runs (12 repetitions), the intra-item variability was obtained by calculating the standard deviation of the beta maps of twelve repetitions voxel by voxel in a given ROI for each word. Note that this approach gave rise to some extreme outlier beta values (for a similar problem, see Malins et al., 2018, p. 2983). We therefore replaced the extreme values (beta values of a voxel greater than 2.5 standard deviations above or below the mean) in a masked beta map by the mean beta value of all voxels. There was no significant difference between the two groups in the number of outlier voxels that were excluded [DYS = 41878 ± 11903 (SD), CTR = 37264 ± 8103 (SD), *t*(38) = 1.43, *p* = 0.16]. The overall percentage of trimmed outlier voxels was 2.03% for the dyslexic group and 1.98% for the control group. The mean variability was then calculated by averaging all the standard deviations in a given ROI. Finally, the mean variability of each subject was calculated by averaging the mean variability of all the words. The mean intra-word variability of two groups in each ROI is shown in Figure 8. Potential group differences were assessed using a two-sample *t*-test for each ROI (*p*-values were corrected for multiple comparisons using FDR). The results showed no significant differences between two groups for intra-word variability in each ROI (all *p*s > 0.05).

Interestingly, intra-word variability seemed smaller in left FG than all other regions. To test for this, we conduced paired *t*-tested of left FG variability against all other ROIs. For the control group, the activity in left FG was significantly less variable than in all the other ROIs (all *p*s < 0.01, corrected for multiple comparisons) except right FG (*p* = 0.29). For the dyslexic group, left FG was significantly less variable than activity in the localizer, bilateral extrastriate cortex, bilateral IFG, bilateral AG, and right SMG (all *p*s < 0.01, corrected for multiple comparisons), but it was not significantly different from right FG and left SMG (*p*s > 0.1).

### Multivariate pattern analysis

#### Representational similarity analysis

**FIGURE 9:**
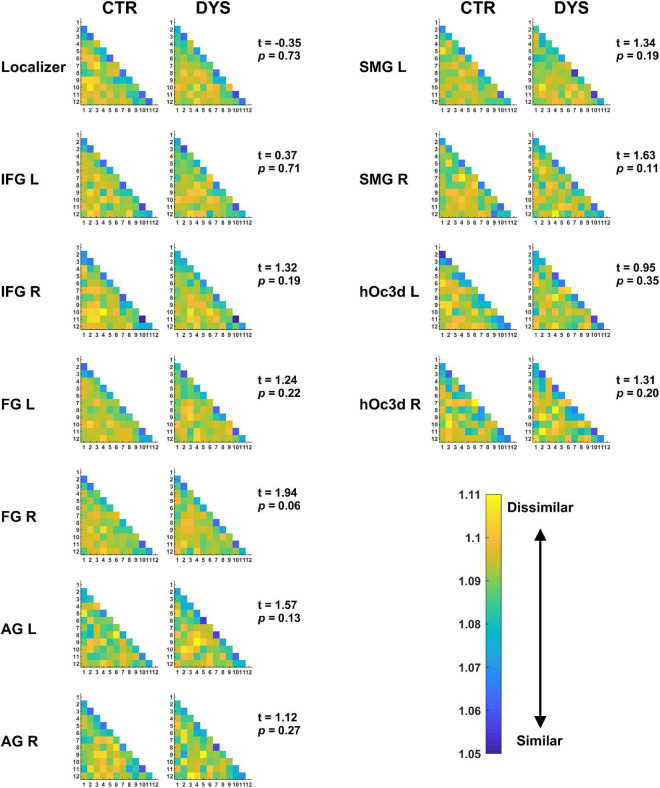
The mean RDMs for repeated words in each ROI. The values in the matrix show the dissimilarity between each pair of repetitions (the higher the value, the lower the similarity). Potential group differences were assessed with a two-sample *t*-test (see *t* and *p*-values). DYS: dyslexic readers, CTR: typical readers, IFG: inferior frontal gyrus, FG: fusiform gyrus, AG: angular gyrus, SMG: supramarginal gyrus, hOc3d: dorsal extrastriate cortex, L: left hemisphere, R: right hemisphere.

For the representational dissimilarity matrix (RDM) analyses, we calculated the RDM values across the 12 repetitions in a given ROI for each word. The mean RDM of each subject was computed by averaging the RDMs of all the words. The mean RDM of word repetitions in each ROI of two groups is displayed in Figure 9. For the statistical group analysis, the mean dissimilarity value for each subject was obtained by averaging all RDM values (i.e., the lower triangle of the matrix leaving out the diagonal). A two-sample *t*-test was used to assess the difference of dissimilarity in RDM between the dyslexic and control groups for each ROI (*t*-values and *p*-values of each ROI are shown in Figure 9). As above, the *p*-values were corrected for multiple comparisons using FDR. The results showed that there were no significant differences in the RDMs between the two groups in any of the ROIs (all *p*s > 0.05).

#### Support vector machine classification of words and hash marks

**FIGURE 10:**
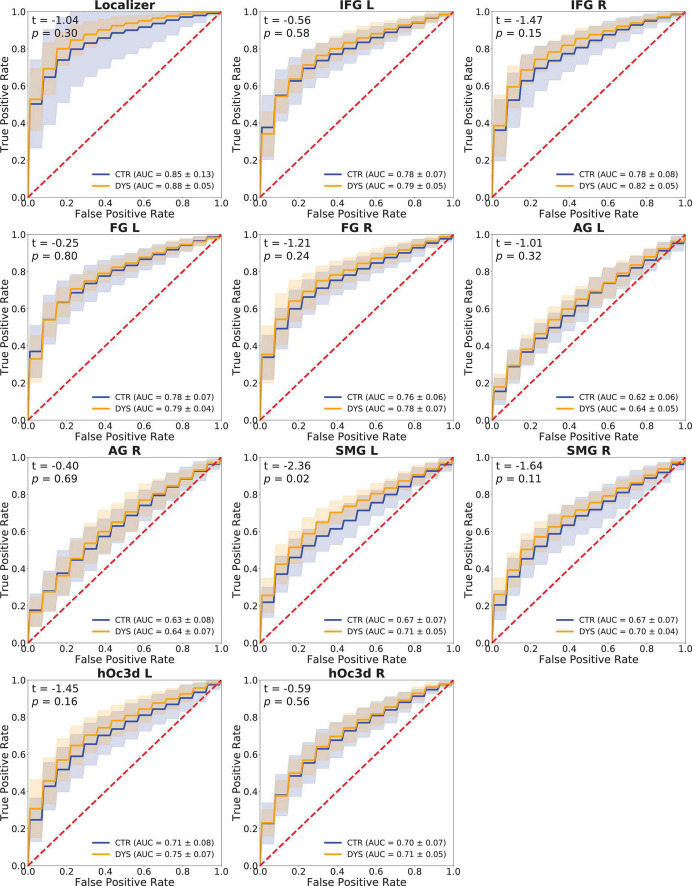
Classification performance (ROC curves) of a Support Vector Machine classifier in each ROI. The mean ROC scores of the two groups are shown in the bottom right corner, *t*-values and *p*-values of a two-sample *t*-test are shown in the upper left corner, the shaded areas correspond to ± 1 SD of the ROC curve. DYS: dyslexic readers, CTR: typical readers, IFG: inferior frontal gyrus, FG: fusiform gyrus, AG: angular gyrus, SMG: supramarginal gyrus, hOc3d: Dorsal extrastriate cortex, L: left hemisphere, R: right hemisphere.

A Support Vector Machine (SVM) classifier was used to classify words and hash marks (see section “Materials and methods”). Classification performance was measured through the ROC-AUC score. The *p*-values of the permutation test were less than 0.05 in all the ROIs for most subjects. It indicates that the classifier was able to classify words and hash marks for most subjects with high accuracy. The ROC curves and mean ROC scores of the two groups in each ROI are displayed in Figure 10.

In order to compare the ROC-AUC scores of the two groups, a two-sample *t*-test was performed (*t*-values and *p*-values of each ROI are shown in Figure 10). It can be seen that the accuracy for the dyslexic group was significantly higher than that of the control group in left SMG (*p* = 0.02). No significant group differences were obtained in the other ROIs.

## Discussion

The goal of the present study was to put the neural noise hypothesis to a direct test by investigating whether there was any evidence for excessive neural noise in adults with dyslexia when neural noise was equated with greater variability in the behavioral and neural responses to repeated presentations of the same stimulus (Hancock et al., 2017). We had participants read aloud words in an MRI scanner and the words were repeated 12 times across four runs intermixed with hash marks, which provided the baseline condition.

The behavioral and univariate results can be summarized as follows. First, the reading level assessment and the reading aloud data in the scanner clearly showed that our university students with dyslexia performed more poorly on all reading measures than the control group (weaker ARHQ scores, weaker fluency in a standardized reading test and slower RTs in reading aloud). The effect sizes of the differences on the standardized tests varied between 2.1 and 3.5 standard deviations below the mean of the controls, which clearly confirms that reading performance in our group of adult dyslexic readers was still in a pathological range and this was true even when compared to normative adult samples (Fichten et al., 2014; Cavalli et al., 2017a). Thus, despite being university students, our sample of adult dyslexic readers read significantly more slowly than controls. Slow reading is a hallmark of DD in adults (Pennington et al., 1990; Lefly and Pennington, 2000; Cavalli et al., 2017b). Second, both groups showed a significant RT decrease across repetitions. However, the repetition effect was not different for the two groups, which goes against a key prediction of the neural noise hypothesis that dyslexic readers should benefit less from repetition than controls. A similar finding was reported by Pugh et al. (2008) who showed significant repetition effects but no interaction between the effects of group and repetition in a word reading paradigm. Third, in the univariate analyses, there was clear evidence for significant repetition effects (neural adaptation) in left FG in adults with dyslexia that was not different to that of typical readers. The same regions also showed significant lexicality effects in dyslexics that were not different to those of controls. Similar findings have been reported by Beach et al., 2022a,b who found repetition effects in adult dyslexia that were quantitatively and qualitatively not different from those of typically developing readers.

The strongest test of the neural noise hypothesis was the intra-item variability analysis (Garrett et al., 2010; Malins et al., 2018). Because each item was repeated three times in each of the four runs (12 repetitions), the intra-item variability could be obtained by calculating the standard deviation of the beta values of 12 repetitions voxel by voxel for each word in a given ROI. Although the results showed some interesting variations of intra-item variability across the ROIs (e.g., the smallest variability was obtained in left fusiform gyrus in line with its key role as the visual word form system, see Dehaene and Cohen, 2011), there was absolutely no evidence for greater variability in the neural response to repeated words in adults with dyslexia.

Given that multiple levels of representation are involved in reading single words (visual representations of letter shape, orthographic representations of letter identity and order, phonological representations of the word’s pronunciation, and semantic representations of its meaning) and they are distributed over a large reading network (Hoffman et al., 2015), we used two multivariate pattern analyses (RSA and SVM classification) that are more sensitive to the distributed nature of the information than our previous analyses (Fischer-Baum et al., 2017). The key prediction for the RSA analysis was that if neural responses to repeated words were noisier, then representational similarity across repeated words should be weaker. Although we found greater representational similarity for word repetitions that occurred with the same run than between different runs, there was again no evidence for noisier neural representations for adult dyslexic readers than controls. In our final test of the neural noise theory, we used a state-of-the art classification algorithm. In this analysis, we no longer looked at variability or similarity across repeated items, but we simply let the classifier do the classification on words vs. Hash marks on the basis of the distributed neural patterns in the data that are present in various ROIs. If the neural responses to words were noisier in adults with dyslexia, the classifier should perform more poorly for adults with dyslexia. Although group mean classification performance was good with AUC-ROC scores between 0.62 and 0.88 depending on the ROI, there were again no differences between the two groups except for superior classification of adult dyslexic readers over controls in left supramarginal gyrus. The supramarginal gyrus belongs to the dorsal route involved in phonological decoding. Superior classification performance of adult dyslexics in that ROI might suggest that they still rely to a greater extent on the less automatized dorsal route than the ventral route when reading words aloud.

A potential problem of our classification null effect is the fact that the word-hash mark classification might have been too coarse to reveal subtle differences in neural representation of word representations. That is, the differences between words and hash marks might be so big that classification performance would not be affected even if word representations were noisier in adults with dyslexia. A stronger test would have been to compute the classification of one word (all its presentations) against another word (all its presentations). However, we had too few presentations of each word to conduct this analysis. Another potential problem is that hash marks did not require a reading aloud response whereas word did. Thus, the classifier could have exploited differences in articulatory output processes to make successful classifications. However, if this were the case, we should have obtained better classification performance in Broca’s area than in fusiform gyrus, which was not the case. In fact, there is little reason to believe that articulatory output processes should affect neural activation in fusiform gyrus.

Taken together, we found no evidence for increased neural noise in adults with dyslexia as defined by greater variability in the behavioral and neural responses to repeated presentations of the same stimulus. Our findings contrast with those of Perrachione et al. (2016) who found less neural adaptation in dyslexic adults for repeated words than for controls. However, the neural adaptation paradigm is very different from our paradigm because, in the neural adaptation paradigm, a single item (e.g., the word “bank”) is presented eight times in a row and participants passively viewed the words. In our paradigm, words were repeated 12 times but they were intermixed with other words and hash marks and participants were asked to make an active response. It is clearly the case that our paradigm is less well suited to measure neural adaptation than that of Perrachione et al. (2016) but more work is needed to fully understand the differences between neural adaptation and repetition paradigms (Pugh et al., 2008).

At first sight, our results seem to be inconsistent with the findings of Malins et al. (2018) who showed that trial-by-trial activation variability in the left IFG pars triangularis was associated with reading skills in a sample of school-aged children. However, in their study, the correlation was positive with greater levels of neural variability being associated with better reading skills. While we did not find greater variability for typically developing readers either, it is worth noting that their results are opposite to the predictions of the neural noise hypothesis. Indeed, the authors suggest that neural variability could be beneficial in developing readers.

One explanation for why we might not have seen neural noise effects could be that the repetition of words, which was our experimental “trick” to study neural noise (variability across repetitions), actually had the opposite effect of “cleaning-up” short-term memory representations for repeated items. Thus, the massive repetition might have rendered the task too easy to tap differences in the quality of underlying representations. This is in line with the finding of Pugh et al. (2008) who showed that after only three repetitions of the same words, the left-hemisphere reading network showed a normal response to written words in dyslexic participants. Although we cannot exclude this possibility, it is worth pointing out that our adults with dyslexia still showed slower reading aloud performance than controls even after 12 repetitions. One obvious shortcoming of the present study is that our dyslexic participants were university students, which might have compensated for their lower-level orthographic and phonological deficits by using context or higher level-linguistic information (Cavalli et al., 2017b,c). Repetition might be one of the contextual factors that is used strategically by adult dyslexic readers to compensate for their persistent low-level orthographic processing deficits. However, while it is easy to see how such compensation strategies can improve reading performance, it is less obvious to see how reading compensation could alleviate neural noise. It would be important to do a similar study with children and contrast neural adaption and neural noise paradigms. Clearly, more work is needed to put this exciting hypothesis to further test.

## Data availability statement

The raw data supporting the conclusions of this article will be made available by the authors, without undue reservation.

## Ethics statement

The studies involving human participants were reviewed and approved by the Comité de Protection des Personnes (CPP). The patients/participants provided their written informed consent to participate in this study.

## Author contributions

JZ and EC designed the study. VC and EC collected the data. YT and VC analyzed the data. J-LA gave advice on data analysis and interpretation. YT wrote the first draft of the manuscript. JZ, VC, and J-LA contributed to several revisions of the first manuscript. All authors contributed to the article and approved the submitted version.

## Footnotes

1
https://www.mathworks.com/help/wavelet/ref/waveletsignaldenoiser-app.html

2
https://www.fil.ion.ucl.ac.uk

3
https://www.nitrc.org/projects/wfu_pickatlas/

4
http://marsbar.sourceforge.net/faq.html#how-do-i-extract-percent-signal-change-from-my-design-using-batch

5
https://github.com/lrkrol/permutationTest

6
https://nilearn.github.io
