# Supplementary material for: Is there evidence for a noisy computation deficit in developmental dyslexia?
Source: Front Hum Neurosci. 2022 Sep 30;16:919465. doi: 10.3389/fnhum.2022.919465 (PMC9561132; doi:10.3389/fnhum.2022.919465)
Supplement: Supplementary file 1 [file Data_Sheet_1.pdf]

Supplementary Material

1 Whole-brain Analysis Results

Supplementary Table 1. The extent, t-values and coordinates in Talairach Space of areas that significantly activated for words > hashmarks in CTR group at an uncorrected voxel-wise statistical threshold of $p < 0.001$ and a cluster-wise threshold of $q < 0.05$ with FDR correction.

**Brain area**	**Extent**	**T values**	**Coordinates**		
			**x**	**y**	**z**
R Cerebellum (VI)	1802	10.810	33	-54	-32
R Cerebellum (VIII)	1802	9.545	20	-66	-52
R Cerebellum (VI)	1802	7.999	-20	-59	-27
R Superior Temporal Gyrus	2794	10.734	63	-11	6
R Rolandic Operculum	2794	9.165	53	-6	23
R Middle Temporal Gyrus	2794	8.675	55	-29	-2
L Superior Temporal Gyrus	3107	10.423	-60	-9	3
L Postcentral Gyrus	3107	9.513	-53	-11	23
L Thalamus	3107	9.135	-13	-14	8
L Cerebellum (VIII)	199	8.052	-20	-66	-55
L IFG (p. Orbitalis)	41	6.136	-38	29	1
L Hippocampus	42	6.032	-18	-29	-7
R Lingual Gyrus	107	5.971	20	-61	3
L Cerebellum (IV-V)	107	5.424	-5	-56	-2
R Posterior-Medial Frontal	81	5.875	3	7	68
L Inferior Parietal Lobule	102	5.810	-40	-44	36
L Angular Gyrus	102	4.571	-30	-61	38
L Brainstem	152	5.591	-13	-29	-32
R Brainstem	54	5.529	13	-34	-35
R ParaHippocampal gyrus	54	3.920	28	-21	-30
R Cerebellum (Crus 1)	46	5.461	10	-84	-17
L Superior Parietal Lobule	27	5.444	-15	-74	61
L Cingulum Ant	58	5.270	0	17	31

R IFG (p. Orbitalis)	71	5.200	53	32	-2
R ParaHippocampal gyrus	27	4.968	20	2	-30

Note. L: left hemisphere, R: right hemisphere.

Supplementary Table 2. The extent, t-values and coordinates in Talairach Space of areas that significantly activated for words > hashmarks in DYS group at an uncorrected voxel-wise statistical threshold of $p < 0.001$ and a cluster-wise threshold of $q < 0.05$ with FDR correction.

**Brain area**	**Extent**	**T values**	**Coordinates**		
			**x**	**y**	**z**
L Putamen	3075	15.397	-28	-19	3
L Precentral Gyrus	3075	14.290	-45	-9	28
L Superior Temporal Gyrus	3075	11.756	-48	-16	6
R Superior Temporal Gyrus	2426	14.153	50	-9	26
R Pallidum	2426	9.465	30	-16	1
R Precentral Gyrus	2426	8.721	50	-9	53
L Cerebellum (VI)	762	11.579	-13	-61	-17
R Cerebellum (VI)	762	9.829	13	-61	-20
R Thalamus	190	7.886	15	-14	1
L Cerebellum (IV-V)	27	6.599	-20	-29	-22
L ParaHippocampal Gyrus	51	6.265	-18	-11	-27
L Medial Temporal Pole	51	4.586	-33	4	-30
L Posterior-Medial Frontal	28	5.975	-3	2	63
L Median cingulate	32	5.950	-5	-11	38
R Cerebellum (VIII)	76	5.822	10	-66	-42
L Cerebellum (VIII)	76	5.622	-13	-66	-45
R Temporal Pole	23	5.756	40	7	-20
R Mid Orbital Gyrus	68	5.296	10	42	-5
R Calcarine Gyrus	123	5.167	5	-84	8
R Lingual Gyrus	56	5.119	13	-49	6
L Lingual Gyrus	22	4.914	-10	-54	8
R ParaHippocampal gyrus	29	4.804	18	-9	-25

Note. L: left hemisphere, R: right hemisphere.

Supplementary Table 3. The extent, t-values and coordinates in Talairach Space of areas that significantly activated for words repetition in CTR group at an uncorrected voxel-wise statistical threshold of $p < 0.001$, and a cluster-wise threshold of $q < 0.05$ with FDR correction.

Brain area	Extent	T values	Coordinates		
			x	y	z
L Precentral Gyrus	162	9.967	-43	-4	48
R Lingual Gyrus	617	9.379	25	-94	-10
R Inferior Temporal Gyrus	617	5.434	40	-66	-2
R Middle Occipital Gyrus	617	4.382	38	-91	8
L Lingual Gyrus	834	8.987	-20	-96	-12
L Cerebelum (Crus 1)	834	7.596	-40	-74	-15
L Inferior Temporal Gyrus	834	5.441	-45	-49	-12
L Middle Temporal Gyrus	100	7.534	-45	-46	11
L Middle Temporal Gyrus	100	4.372	-58	-31	6
R Superior Temporal Gyrus	57	6.805	50	-19	-5
L Median cingulate	66	6.553	-10	12	43
L Insula Lobe	30	6.166	-35	14	11
R Precentral Gyrus	32	5.583	48	-4	43
L Median cingulate	35	4.891	0	-14	43
R Supplementary motor area	29	4.490	13	4	46

Note. L: left hemisphere, R: right hemisphere.

Supplementary Table 4. The extent, t-values and coordinates in Talairach Space of areas that significantly activated for words repetition in DYS group at an uncorrected voxel-wise statistical threshold of $p < 0.001$, and a cluster-wise threshold of $q < 0.05$ with FDR correction.

Brain area	Extent	T values	Coordinates		
			x	y	z
R Lingual Gyrus	450	10.645	20	-99	-10
L Inferior Occipital Gyrus	507	8.861	-25	-96	-15

L Middle Occipital Gyrus	507	5.690	-30	-96	6
L Fusiform Gyrus	507	4.860	-35	-69	-7
L Cerebellum (Crus 1)	157	6.176	-40	-59	-25
L Cerebellum (Crus 1)	157	4.047	-33	-81	-22
L Insula Lobe	29	6.148	-43	9	8
R Precentral Gyrus	43	5.566	48	-6	38

Note. L: left hemisphere, R: right hemisphere.

2 Univariate Region of Interest (ROI) Analysis

2.1 ROI Definition

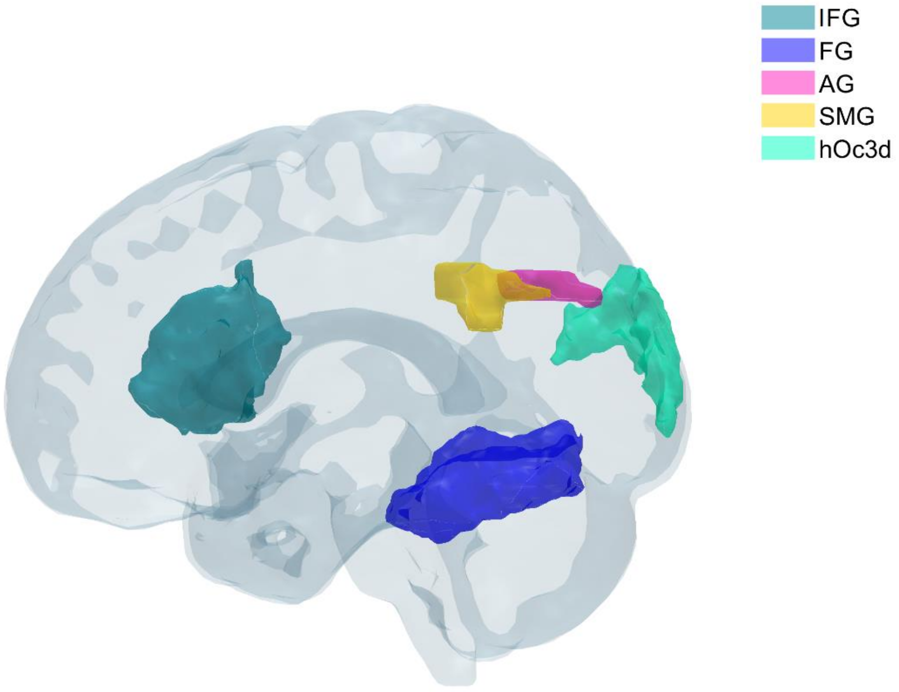

Supplementary Figure 1. 10 predefined ROI (only 5 ROIs on the left side of the brain are shown) were selected in this analysis: left and right inferior frontal gyrus (IFG), left and right fusiform gyrus (FG), left and right angular gyrus (AG), left and right supramarginal gyrus (SMG) and left and right hOC3d (dorsal extrastriate cortex). Here for visualization, the ROIs are overlayed on a standard human brain (Montreal Neurological Institute cerebral atlas) but for the univariate ROI analysis, they were extracted for each subject in the native space.

2.2 Results on Lexicality effect (first run)

Supplementary Table 5. The table presents the effect size and p Values (p) of the lexicality contrast compared to the null hypothesis for the first run. The values highlighted indicate significant effects with or without correcting for multiple comparisons (FDR or Bonferroni correction; Unc means uncorrected). The color bar specifies the level of significance.

	**Dys**				**Ctr**			
ROI	effectsize	pUnc	pFDR	pBonf	effectsize	pUnc	pFDR	pBonf
IFG L	1.09	0.001	0.015	0.015	1.30	0.000	0.005	0.005
IFG R	0.71	0.016	0.055	0.165	1.13	0.001	0.005	0.010
FG L	0.85	0.007	0.037	0.075	0.50	0.059	0.198	0.595
FG R	0.12	0.354	0.616	3.543	-0.71	0.986	0.996	9.855
AG L	-1.27	1.000	1.000	9.995	-0.82	0.993	0.996	9.930
AG R	-1.19	0.999	1.000	9.990	-0.85	0.996	0.996	9.960
SMG L	-0.18	0.714	1.000	7.141	-0.02	0.510	0.996	5.102
SMG R	-0.28	0.808	1.000	8.081	-0.84	0.995	0.996	9.945
hOc3d L	0.30	0.166	0.415	1.659	0.03	0.461	0.996	4.608
hOc3d R	0.12	0.370	0.616	3.698	-0.14	0.670	0.996	6.697

	**Dys > Ctr**			
ROI	effectsize	pUnc	pFDR	pBonf
IFG L	-0.24	0.777	0.777	7.771
IFG R	-0.20	0.746	0.777	7.461
FG L	0.29	0.174	0.581	1.744
FG R	0.60	0.033	0.330	0.330
AG L	-0.17	0.701	0.777	7.011
AG R	-0.11	0.617	0.777	6.172
SMG L	-0.13	0.668	0.777	6.682
SMG R	0.39	0.104	0.522	1.044
hOc3d L	0.21	0.260	0.595	2.599
hOc3d R	0.18	0.297	0.595	2.974

$p < .05$
$p < .01$
$p < .001$

2.3 Results on Deactivation effect (first run)

Supplementary Table 6. The table presents the effect size and p Values (p) of the deactivation contrast compared to the null hypothesis for the first run. The values highlighted indicate significant effects with or without correcting for multiple comparisons (FDR or Bonferroni correction; Unc means uncorrected). The color bar specifies the level of significance.

	**Dys**				**Ctr**			
ROI	effectsize	pUnc	pFDR	pBonf	effectsize	pUnc	pFDR	pBonf

IFG L	-0.48	0.936	0.996	9.360	-0.25	0.763	0.913	7.626
IFG R	-0.82	0.996	0.996	9.955	-0.30	0.822	0.913	8.221
FG L	0.88	0.005	0.050	0.050	0.50	0.057	0.285	0.570
FG R	0.70	0.018	0.090	0.180	0.90	0.003	0.030	0.030
AG L	0.13	0.350	0.875	3.498	-0.24	0.754	0.913	7.536
AG R	0.22	0.258	0.860	2.579	-0.53	0.941	0.941	9.410
SMG L	0.02	0.467	0.934	4.668	-0.28	0.801	0.913	8.011
SMG R	-0.23	0.759	0.996	7.586	0.00	0.506	0.913	5.062
hOc3d L	-0.51	0.942	0.996	9.415	0.20	0.273	0.911	2.734
hOc3d R	-0.72	0.985	0.996	9.845	0.06	0.434	0.913	4.343

	Dys > Ctr			
ROI	effectsize	pUnc	pFDR	pBonf
IFG L	-0.14	0.666	0.914	6.662
IFG R	-0.20	0.731	0.914	7.311
FG L	0.34	0.145	0.612	1.454
FG R	0.03	0.469	0.914	4.693
AG L	0.23	0.218	0.612	2.179
AG R	0.47	0.074	0.612	0.740
SMG L	0.22	0.245	0.612	2.449
SMG R	-0.17	0.705	0.914	7.046
hOc3d L	-0.51	0.943	0.968	9.425
hOc3d R	-0.58	0.968	0.968	9.680

p < .05
p < .01
p < .001

2.4 Results on Lexicality effect (all runs)

Supplementary Table 7. The table presents the effect size and p Values (p) of the lexicality contrast compared to the null hypothesis for all runs. The values highlighted indicate significant effects with or without correcting for multiple comparisons (FDR or Bonferroni correction; Unc means uncorrected). The color bar specifies the level of significance.

	Dys				Ctr			
ROI	effectsize	pUnc	pFDR	pBonf	effectsize	pUnc	pFDR	pBonf
IFG L	0.99	0.001	0.005	0.010	1.73	0.000	0.002	0.005
IFG R	1.40	0.000	0.005	0.005	1.30	0.000	0.002	0.005
FG L	0.58	0.037	0.092	0.370	0.41	0.097	0.325	0.975
FG R	-0.18	0.714	0.956	7.136	-0.86	0.996	0.999	9.960
AG L	-0.23	0.765	0.956	7.651	-0.79	0.994	0.999	9.940
AG R	-0.41	0.893	0.969	8.931	-0.71	0.985	0.999	9.845
SMG L	-0.10	0.626	0.956	6.257	-0.01	0.521	0.999	5.207
SMG R	-0.59	0.969	0.969	9.685	-0.91	0.999	0.999	9.990
hOc3d L	0.95	0.002	0.007	0.020	-0.02	0.509	0.999	5.087
hOc3d R	-0.17	0.710	0.956	7.101	-0.21	0.734	0.999	7.341

	Dys > Ctr			
ROI	effectsize	pUnc	pFDR	pBonf
IFG L	-0.34	0.868	0.868	8.676
IFG R	0.27	0.199	0.399	1.994
FG L	0.17	0.309	0.441	3.088
FG R	0.49	0.068	0.342	0.685
AG L	0.29	0.182	0.399	1.819
AG R	0.20	0.253	0.422	2.534
SMG L	-0.08	0.601	0.668	6.012
SMG R	0.41	0.105	0.351	1.054
hOc3d L	0.53	0.047	0.342	0.475
hOc3d R	0.01	0.483	0.603	4.828

p < .05
p < .01
p < .001

2.5 Results on Deactivation effect (all runs)

Supplementary Table 8. The table presents the effect size and p Values (p) of the deactivation contrast compared to the null hypothesis for all runs. The values highlighted indicate significant effects with or without correcting for multiple comparisons (FDR or Bonferroni correction; Unc means uncorrected). The color bar specifies the level of significance.

	Dys				Ctr			
ROI	effectsize	pUnc	pFDR	pBonf	effectsize	pUnc	pFDR	pBonf
IFG L	-0.29	0.805	0.958	8.051	-0.97	0.999	0.999	9.985
IFG R	-0.37	0.862	0.958	8.621	-0.83	0.995	0.999	9.950
FG L	0.75	0.016	0.148	0.160	0.40	0.105	0.488	1.054
FG R	0.59	0.036	0.148	0.360	0.33	0.146	0.488	1.464
AG L	0.56	0.044	0.148	0.445	-0.07	0.569	0.812	5.687
AG R	0.29	0.181	0.452	1.809	-0.59	0.962	0.999	9.620
SMG L	0.05	0.425	0.709	4.253	-0.02	0.505	0.812	5.052
SMG R	0.08	0.398	0.709	3.978	0.12	0.348	0.812	3.478
hOc3d L	-0.27	0.794	0.958	7.936	0.37	0.130	0.488	1.299
hOc3d R	-0.59	0.965	0.965	9.645	0.02	0.480	0.812	4.798

	Dys > Ctr			
ROI	effectsize	pUnc	pFDR	pBonf
IFG L	0.51	0.054	0.192	0.545
IFG R	0.30	0.174	0.435	1.739
FG L	0.18	0.291	0.502	2.914
FG R	0.16	0.301	0.502	3.013
AG L	0.51	0.057	0.192	0.575
AG R	0.63	0.026	0.192	0.260
SMG L	0.05	0.438	0.625	4.378

SMG R	-0.04	0.533	0.666	5.327
hOc3d L	-0.43	0.914	0.914	9.135
hOc3d R	-0.37	0.877	0.914	8.766

$p < .05$
$p < .01$
$p < .001$
